# Editorial: Neuroinflammatory and oxidative/nitrosative pathways in neuropsychiatric and neurological diseases and their possible neuropharmacological regulation, volume I

**DOI:** 10.3389/fphar.2022.1033281

**Published:** 2022-09-16

**Authors:** Marta P. Pereira, Borja García-Bueno, Javier R. Caso

**Affiliations:** ^1^ Departamento de Biología Molecular, Centro de Biología Molecular “Severo Ochoa” (UAM-CSIC), Madrid, Spain; ^2^ Instituto Universitario de Biología Molecular, IUBM, Universidad Autónoma de Madrid, Madrid, Spain; ^3^ Departamento de Farmacología y Toxicología, Facultad de Medicina, Universidad Complutense de Madrid (UCM), Instituto de Investigación Sanitaria Hospital 12 de Octubre (Imas12), Instituto Universitario de Investigación en Neuroquímica (IUIN-UCM), Madrid, Spain; ^4^ Centro de Investigación Biomédica en Red de Salud Mental, Instituto de Salud Carlos III (CIBERSAM, ISCIII), Madrid, Spain

**Keywords:** neuropsychiatric diseases, neurological diseases, neuroinflammation, oxidative/nitrosative stress, neuropharmacology

There is a crucial necessity to identify new pathophysiological pathways and therapeutic targets for neurological and neuropsychiatric diseases, as they are rising as leading causes of death, disability, and overall disease burden (Murray et al., 2012). Bearing in mind the potential role of neuroinflammation and the subsequent oxidative/nitrosative damage in these illnesses (Caldwell et al., 2020) the development of new drugs based in the modulation of the immune response is a necessary approach for future therapeutic interventions that deserves further consideration from the standpoint of the neuropharmacology. Thus, the goal of this Research Topic is to offer a view of the current developments in the neuroimmune pharmacology realm for the neurological and neuropsychiatric diseases.

This Research Topic offers an overview of the neuroinflammatory and oxidative/nitrosative pathways in neuropsychiatric and neurological diseases through 11 articles written by 80 authors. This assemblage comprises one mini review, one opinion article, one brief research report, and eight original research papers.

Leite et al. reviewed the role of ouabain, a cardiac steroid known to modify certain immune responses, as a modulator of the neutrophilic neuroinflammation. The authors highlight the reduction of the proinflammatory enzymes activation, and the lower levels of the interleukin (IL)-1 caused by ouabain in the CNS. Authors concluded that ouabain and its receptor (NKA) might be of interest for developing new strategies in the fight against diseases with a neuroinflammatory component.

In an opinion article, Coppens et al. bring to mind that microglia- or astrocyte-derived tryptophan catabolites (TRYCAT) have neuromodulatory effects on the NMDA receptor. Hence, this opinion piece hypothesizes that TRYCAT might link immune responses to clinical symptomatology in psychotic and mood disorders. Additionally, authors discuss the impact of sample heterogeneity, methodological consistency, and validity on study data, putting forward conceptual and methodological advices for future research as well as suggestions for appropriate yet to come research possibilities.

Caso et al. aimed to study immune factors and related risk paths in adult female patients with eating disorders (ED) as well as to find psychological factors prompting the inflammatory response. An important feature of this article is the naïve nature of the sample, meaning that none of the patients was taking medication at the time of assessment. Patients shown higher proinflammatory and oxidative/nitrosative stress parameters than healthy controls. Besides, correlations between impulsiveness and depressive symptomatology and some inflammatory parameters were also identified. Overall, the main conclusion reached was that inflammatory factors could be considered as potential therapeutic targets in ED.

Fu et al. looked into valproic acid-resistant epilepsy, a condition in which most patients present an inflammatory response and local hypoxia. This study identified that the hypoxia-inducible factor (HIF)-1α (a critical effector parameter of hypoxia and inflammation) was overexpressed in mice with valproic acid-resistant epilepsy and regulated the expression of some proinflammatory mediators through the induction of the polarization of microglia from the M2 phenotype to M1 phenotype. Authors were also able to conclude that miR-221-3p/HIF-1α is a vital component in pathogenesis of valproic acid-resistant epilepsy, representing a potential therapeutic antiseizure target.

A classical model for the study of neuroinflammation is the injection of bacterial lipopolysaccharide (LPS) (Zhao et al., 2019). Building in this model, Piovan et al. investigated the effect of pre- and post-treatment with an extract of spirulina in an *in vitro* model of LPS-induced microglia activation, observing a downregulation of important LPS-induced proinflammatory factors and an upregulation of antioxidant mechanisms, showing that the extract of spirulina can be useful in the control of microglia activation and neuroinflammatory processes.

Neuropathic pain (NP) is a chronic condition that can be caused by nervous system damage and studies have shown that inflammation and oxidative stress are important processes in the pathological process of this kind of pain (Costigan et al., 2009). Yang et al. assessed the variations in reactive oxygen species levels, lipid peroxidation, and antioxidant systems in different rodent models of pain and the effects of higenamine (a plant-based alkaloid with anti-inflammatory and antioxidant effects) on these parameters. Higenamine improved the oxidative stress and the inflammation induced by the pain models, warranting further research as a potential drug for the treatment of NP.

Parkinson’s disease (PD) is the second most frequent neurodegenerative disease in the world and the most common movement disorder for which there is presently no cure. For that reason, more tools for its study are required. In this sense, cell replacement therapy is a potential treatment for PD. Nelke et al. characterized and transplanted a line of human neural stem cells in middle-aged Parkinsonian mice. Their cell replacement therapy approach prevented motor and non-motor impairments.

Jin et al. studied the effects of metformin, a drug employed in the treatment of type 2 diabetes with previously described anti-inflammatory effects, in a mouse model of early brain injury after subarachnoid hemorrhage. Metformin was neuroprotective by modulating the production of proinflammatory factors induced by the inflammasome NLRP3 and the activation of microglia.

Deng et al. have studied the roles of pyroptosis-related genes in major depressive disorder, as pyroptosis has been found as an inflammatory form of programmed cell death. Authors were able to split depression cases into two distinct pyroptosis subtypes with dissimilar immune and biological traits. Their results indicate that pyroptosis may perform a valuable part in depression providing new insights into its diagnosis and pathophysiology.

Garcia-Partida et al. investigated the actions of mangiferin (a polyphenolic compound abundant in the leaves of *Mangifera indica* that has strong antiinflammatory and antioxidant qualities) comparing them with the actions of risperidone (an antipsychotic drug) in a rat model of schizophrenia. Their article included behavioral and neuroimaging studies and the measurement of oxidative/inflammatory and antioxidant mediators. Authors suggest that mangiferin might be a possible therapeutic or preventive add-on strategy to improve the clinical expression of schizophrenia in adulthood.

Finally, Montes et al. employed rats and post-mortem human frontal cortex and cerebellum to study the effects of the combination of chronic alcohol consumption and thiamine deficiency on the innate immune system and their specific contribution to the pathogenesis of Wernicke’s encephalopathy (WE). Their findings offer data, both in the animal model and the human postmortem brain, of the upregulation of the TLR4/MyD88 proinflammatory pathway in WE related to alcohol consumption.

In conclusion, this Research Topic confirms once again the importance of neuroinflammation and oxidative stress as key players in the etiology of neurological and neuropsychiatric diseases. The effects of these players, not only at the biochemical but also at a behavioral level, support the efforts in the search of new modulatory drugs, as they are crucial targets for the development of new pharmacological therapies.
